# Announcements

**Published:** 2015-05-01

## Arthritis Awareness Month — May 2015

May is Arthritis Awareness Month. Arthritis affects an estimated 52.5 million U.S. adults (1), is a common comorbidity among those with multiple chronic conditions, and is a leading cause of disability in the United States (2).

Although physical activity can help reduce joint pain and disability among those with arthritis, only one in 10 persons with arthritis meet HHS physical activity guidelines of 150 minutes of moderate activity per week (3) Walking is a preferred exercise among arthritis patients (4) and has been shown to improve arthritis symptoms, physical function (e.g., walking speed), and quality of life (5). The importance of walking was underscored in a recent report demonstrating that decreased physical function, as documented by walking speed, was related to both all-cause and cardiovascular disease deaths among adults with hip osteoarthritis (6). For those concerned about safely increasing their walking, programs like Walk With Ease (WWE), a 6-week walking program, can help. WWE has been shown to reduce pain and fatigue and increase function, ability, strength, balance, and walking pace among adults with arthritis and is one of several CDC-recommended physical activity interventions (http://www.cdc.gov/arthritis/interventions/physical-activity.html). Increased availability of WWE programs in community settings for adults with arthritis will help them reduce their pain and improve their health.

Information about ways to help manage arthritis is available at http://www.cdc.gov/arthritis. Additional information is available from the Arthritis Foundation (http://www.arthritis.org) and the National Institute of Arthritis and Musculoskeletal and Skin Diseases (http://www.niams.nih.gov).
